# Emergency Physicians at War

**DOI:** 10.5811/westjem.2018.1.36233

**Published:** 2018-03-08

**Authors:** Andrew E. Muck, Melissa Givens, Vikhyat S. Bebarta, Phillip E. Mason, Craig Goolsby

**Affiliations:** * University of Texas Health at San Antonio, Department of Emergency Medicine, San Antonio, Texas; † Uniformed Services University, Department of Military and Emergency Medicine, Bethesda, Maryland; ‡ University of Colorado Denver, Department of Pharmacology, Denver, Colorado; § San Antonio Military Medical Center, Department of Emergency Medicine, San Antonio, Texas

## Abstract

Operation Enduring Freedom (OEF-A) in Afghanistan and Operation Iraqi Freedom (OIF) represent the first major, sustained wars in which emergency physicians (EPs) fully participated as an integrated part of the military’s health system. EPs proved invaluable in the deployments, and they frequently used the full spectrum of trauma and medical care skills. The roles EPs served expanded over the years of the conflicts and demonstrated the unique skill set of emergency medicine (EM) training. EPs supported elite special operations units, served in medical command positions, and developed and staffed flying intensive care units. EPs have brought their combat experience home to civilian practice. This narrative review summarizes the history, contributions, and lessons learned by EPs during OEF-A/OIF and describes changes to daily clinical practice of EM derived from the combat environment.

## INTRODUCTION

Operation Enduring Freedom in Afghanistan (OEF-A) and Operation Iraqi Freedom (OIF) in Iraq, were the first major, sustained conflicts that involved formally trained emergency physicians (EPs) treating larger numbers of war-wounded. OEF-A began in October 2001 and officially completed in December 2014, although U.S. troops remain in Afghanistan as part of Operation Freedom’s Sentinel.^1^ Between 2001 and 2017, there were 2,400 U.S. military fatalities^2^ and 20,048 U.S. military considered wounded in action.^3^ Operation Iraqi Freedom began in March 2003 and transitioned in September 2010 to Operation New Dawn through 2011.^4^ U.S. troops remain in Iraq at this time. Between 2003 and 2017, 4,520 U.S. military fatalities occurred^5^ and 31,956 were wounded in OIF.^6^ These numbers do not include civilian contractors and local citizens who were patients routinely cared for during the conflicts.

EPs worked in all settings during the conflicts from point-of-injury to transport to tertiary care. The roles of EPs were more limited at the beginning of the conflicts and expanded to ultimately include direct emergency care, unique missions, and leadership. EPs are now the most frequently deployed medical specialists by percentage in the U.S. Army and U.S. Air Force (USAF) and the second most in the U.S. Navy.^7^ EPs’ ability to prioritize emergency conditions, thrive in chaotic and resource-constrained environments, and remain cognitively and clinically flexible proved valuable in combat.

This article shares the story of military EPs by highlighting the indispensable roles they played throughout these recent combat operations.

## MILITARY EMERGENCY PHYSICIANS

The military environment of EPs is complex. Each year changes occur rapidly in combat training requirements, retention, tasking for combat deployments, as well as in the policies of the different military branches. For example, in the USAF for the past three years, there have been approximately 34 EPs trained each year. On average, 20 of those 34 have trained each year at military programs and 14 have trained at civilian programs (a process called “civilian deferment”). EPs who train either in a military program or civilian deferment owe a time commitment to the military. Often, military physicians do not stay past the years owed. Many military EPs choose to return to civilian practice after their initial time commitment is complete, rather than remain in the military for a career of 20 years or more, There is variability in the length of deployments in the different services. For instance, an Army deployment could be 12 months depending upon the circumstances, while an Air Force deployment is typically six months. The frequency of deployment is also variable by service and circumstance, but was often one time period deployed, followed by two time periods at home, often followed by deployment again.^7^ The Navy and Marines similarly have provided strategies for deployment to allow dwell times at home to improve wellness and combat readiness.

The variation in U.S.-based clinical environments is difficult to compare to the diverse experiences EPs confronted during OEF-A/OIF. The following review explores several unique environments and job positions that EPs faced: medical planning, levels (echelons) of care with dramatically varied capabilities, special operations units, and critical care air transport teams.

### Roles in Deployment

#### Settings for deployment and levels of care

The military divides its medical assets into “roles” based on capabilities, and EPs served to some degree in each of these levels (or echelons) of care. Some understanding of the levels of care is important to understand the roles of EPs during OEF-A and OIF. Initially, the injured casualty (self care), fellow soldiers, and medics provide point-of-injury (POI) care. Role 1 facilities, which are attached to small military units, provide emergency first aid, triage, and non-surgical resuscitative care. Role 1 facilities are staffed by one or two physicians or physician assistants and augmented by medics. Role 2 facilities expand this initial capability with additional services, such as dental and limited laboratory capability, and can be combined with a team that provides stabilizing surgical capabilities.^8^ Role 3 facilities are the most robust facilities in a combat zone, and function like trauma centers. Role 3 facilities have subspecialty medical and surgical capability, but do not have all the resources of a U.S.-based Level I trauma center. Role 4 and 5 facilities are outside the combat zone and have progressively more capability as it relates to staff, specialists, services (for example, dialysis).^8^

EPs worked at all levels of care during OEF-A/OIF, and played a robust role in providing en route care as casualties transitioned between these levels. EPs and EMS-subspecialty trained physicians provided medical direction and implemented prehospital education programs that impacted battlefield survival. In addition to serving in all the levels of care above, EPs served in many other unique environments including medical engagement missions with local communities, humanitarian assistance work, advisors to foreign medical systems, and liaison roles with allied governments, among other roles.

#### Clinical Leaders

EPs performed combat support planning at a variety of levels in OEF-A/OIF. EPs served in high-level leadership roles for combat medical units, such as medical group commanders, as well as in military staff and advisory positions. As EPs advanced to military leadership roles, they assisted with the strategic planning of the military’s entire medical support system. While difficult to quantify, EPs significantly impacted military medical preparation, evacuation platforms, and hospital commands during OEF-A/OIF in a way never previously experienced in U.S military history.

**Population Health Research Capsule**

What do we already know about this issue?

Many are aware that emergency physicians (EPs) were deployed to Iraq (OIF) and Afghanistan (OEF-A), but many do not know of all their roles and contributions, nor of the impact on civilian practice.

What was the research question?

This paper reviews the roles of EPs in battle zones and how civilian practice has been affected as a result.

What was the major finding of the study?

The recent conflicts were a major utilization of EPs in war, and civilian practice has been affected.

How does this improve population health?

This narrative review summarizes the history, contributions, and lessons learned by EPs during OIF and OEF, and describes changes to civilian practice derived from the combat environment.

EPs have contributed to the Committee on Tactical Combat Casualty Care (CoTCCC) that developed, promulgated, and refined the Tactical Combat Casualty Care (TCCC) guidelines.^9^ TCCC directs medical care within the unique limitations of a resource-constrained, hostile environment. EPs were themselves educators and trainers for many levels of providers, to include the training of frontline medics. EPs also conducted research that led to improvements in combat casualty care and improved outcomes.

#### Special Operations

EPs served with a variety of special operations units. EPs regularly provided medical care in austere locations during high-risk operations conducted by elite fighting units. National security classification prevents us from knowing the full impact of EPs’ involvement in these missions. However, special operations units routinely request EPs for their diverse skill set and “can do” mindset in challenging circumstances. Even when EPs did not directly participate in combat missions, they supported special operations units through medical planning and training far-forward medics who provide direct patient care.

#### Critical Care Air Transport (CCATT)

Critical Care Air Transport Teams (CCATT), conceived by the USAF in the 1990s and first used in large-scale operations during OEF-A/OIF, allow safe and rapid movement of critically injured and ill patients.^10^ CCATT teams consist of a critical care-qualified physician, a critical care nurse, and a respiratory therapist with an equipment package designed to support three ventilator-dependent patients. CCCAT in particular were involved in intra-theater transport as well as transports of greater length. Common transports of greater length included Iraq or Afghanistan to U.S. airbases/hospitals in Germany. After further procedures or interventions, the patients would then be transported to the U.S. from Germany.

EPs contribute approximately 40% of deployed CCATT requirements and have filled key CCATT leadership and instructor roles.^11^ Physicians are prepared for deployment with two courses focused on providing critical care at altitude, the austere environment of an aircraft, and equipment familiarization. EPs deployed as CCATT physicians faced a challenging case mix with approximately two-thirds of patients having poly-trauma injuries and the remainder with complex medical diagnoses. Among trauma patients, 60% had Injury Severity Scores > 15 and over a quarter had a score > 25. EPs provided complex critical care interventions to these patients, including mechanical ventilation (80%), blood product administration (9%), intracranial pressure monitoring (13%) and vasopressor use (15%).^12^

Various studies commented on the absence of serious problems during transport, such as flights not diverting due to unstable patients and exceptionally few deaths during flight or in the 24-hour time period after flight.^12^,^13^ The mean time from battlefield injury to aircraft launch for the U.S. military hospital in Germany was 28 hours, and 93% of all CCATT patients arrived in Europe within 72 hours of injury. Most patients arrived in the U.S. a few days later. By comparison, it took an average of 45 days to move patients from the battlefield to the U.S. during the Vietnam War.^14^

Analysts credit this rapid transport of critically ill casualties, unprecedented in prior wars, with a marked reduction in mortality.^15^ A 10-year review of Joint Theater Trauma Registry (JTTR) data demonstrated an en route mortality of less than 0.02%, and an overall 30-day mortality of 2.1%.^16^ Building on these OEF-A/OIF successes, CCATT teams have played roles in civilian disaster response including Hurricane Katrina and the 2010 Haiti earthquake, and analysts recommended increasing their use in the future.^17^

#### Roles of Reservists

The conflicts of OEF-A and OIF relied on the National Guard and Reserves to a remarkable degree, to include physician roles. The impact of such deployments led to many EPs being deployed in active duty roles, to include frequent participation in all roles previously described in OEF-A and OIF. The actual numbers of EPs deployed from the National Guard and Reserves is not provided here, but we believe that such EPs provided a great deal of support and their impact should be recognized.

### Unique Patients

EPs treated complex, severely injured poly-trauma patients during OEF-A/OIF. The complexity of injuries of a single patient was notable as the advent of powerful improvised explosive devices (IED) wrought remarkable injury patterns to individuals in vehicles. Dismounted IED injuries were frequently experienced at the height of OEF-A and commonly resulted in amputations. EPs’ contributions to the military medicine team helped more than 97% of injured casualties who reached combat hospitals survive.^18^ Despite a recognition throughout the military that EPs offered a unique contributions and skill set, a review of the literature reveals that few articles highlighted the unique contributions and skill set of EPs.^19^,^20^ Through recognition of the unique patients in the combat environment and how EPs are uniquely suited to treat them, this provides further evidence for contributions of EPs during conflict.

EPs’ role in trauma care certainly deserves emphasis. However, as seen in previous wars, more soldiers during OEF-A/OIF suffered from disease and non-battle injuries (DNBI) than from battlefield injuries.^21^ The ability of an EP to manage such diverse disease conditions from toxic ingestions, environmental exposures, infectious diseases (the rare and the common), psychiatric conditions, obstetric and gynecologic emergencies, and pediatric conditions was repeatedly voiced by command to be invaluable in the deployed settings of Afghanistan and Iraq. EPs’ patients included U.S. soldiers, sailors, airmen, Marines, members of the Coast Guard, allied military members, U.S. federal and contract workers, local national civilians, opposing military members and prisoners of war, third-country national civilians (often contractors hired to work at U.S. bases), and children, among others. EPs’ unique training and experience prepared them to treat the full spectrum of patients, diseases, and injuries encountered in OEF-A/OIF.

### Mass Casualty (MASCAL) Events

Mass casualty (MASCAL) events occurred frequently during OEF-A/OIF, loosely defined by volume of patient numbers and patient requirements that were beyond the normally used resources. Military EPs planned, participated and led during MASCAL events throughout both war theaters. The authors personally responded to multiple MASCALs during their deployments. One author participated in a 45-patient MASCAL, the majority of whom were children, from a suicide bomber in a local park. This presented unique challenges for a military facility equipped to treat adults. Another author responded to multiple MASCALs during a 24-hour long patient surge that resulted in a facility equipped with 10 beds and two EPs treating 60 seriously injured patients. Lessons from these experiences have been applied in the U.S. During the 2009 Fort Hood shooting, combat-experienced EPs contributed to positive outcomes for 30 patients with life-threatening injuries who presented to the base’s small military community hospital.^22^ Specific lessons learned in combat that contributed to the positive outcomes included having an appropriate MASCAL plan, rapid and appropriate adjustments to the plan, positive interactions between physicians, expectation of a second wave, and having a calm approach in a chaotic scenario.

### Knowledge and Skill Translation

EPs returned with combat medical-care experience and skills that were immediately applied and have been disseminating throughout the civilian system. The knowledge and skill translation demonstrates how the combat experiences of EPs has impacted medical care in the non-combat environment and how healthcare is delivered to provide best practices in the domestic world of clinical practice. This was highlighted by Kellermann and Peleg after the Boston bombing as it related to the treatment of bombing victims when they wrote, “Although most health care providers in the United States have never treated a bombing victim, lessons learned by military surgeons, emergency physicians, and nurses in Iraq and Afghanistan are progressively percolating through the trauma care community.”^23^ One example of practice or approach change brought by war includes hemorrhage control, after it was recognized as an important cause of death in the combat environment.^24^ The importance of hemorrhage control advanced the use of tourniquets in the civilian setting. Tourniquet use has been highlighted in such examples as laypersons being employed for tourniquet application^25^ and the national “Stop the Bleed” campaign.^26^ As many providers used hemostatic dressings in the combat setting, they were quick to look for applicable opportunities in the civilian setting. The resuscitation practice of increasing platelets and plasma ratios with packed red blood cells (1:1:1 or 1:1:2) was used in the theater of war and was supported for use in the civilian setting by Holcombe et al.^27^ The use of intraosseous devices was further advanced by many of us who used them frequently in the theater of war.^28^,^29^ Advances in cricothyroidotomy techniques and devices were promulgated as a result of the combat environment.^30^ Of note, many military providers had experience using medications for pain that were used in novel ways and studied during OEF-A/OIF. The synergy of military studies and civilian studies led to the study of such things as intranasal ketamine and ketamine for pain.^31^,^32^

### Future of Military Emergency Physicians

In the immediate time frame, deployments continue with anticipated benefit to the broadening experience of EPs, the advance of emergency medicine, and the knowledge transfer that will occur when EPs bring back lessons learned to the civilian practice environment. This knowledge transfer occurs through publications, educational opportunities between the military and civilian communities and by EPs transitioning out of the military and beginning civilian practice.^33^ An example of civilian trainings directly resulting from the military experience include the TCCC training now used by the National Association of Emergency Medical Technicians. Another example includes the Medical Emergency Response Teams (MERT) borrowed from the military that is now used in civilian training programs.

As to EPs in the military, there is no evidence to expect that the reliance on EPs or the expanded roles will diminish anytime soon. Uniformed Services University (USU) and the military graduate medical education (GME) system continue to train providers with the unique perspectives of military and combat medicine. Combat medicine continues to grow as a unique area of research and training with heavy overlap between EMS and disaster medicine. The role of EPs is expected to continue to grow in the military in the role of leaders/planners. The military has EP contributors and leaders in research in such organizations as the Institute of Surgical Research, Joint Trauma System, and the En Route Care Research Center.

## LIMITATIONS

Multiple attempts were made to obtain specific data that would support some of the assertions made. For instance, we went to great lengths in our attempt to find specific data on such things as the numbers of EPs in leadership roles and EPs deployed within a specific branch of service, given role, or specialty. However, the authors were unable to obtain such data, which limits the strength of the conclusions. The limitations of ill-defined data also limited our discussion on topics such as the expanding role of EPs in combat. Every attempt was made to not overstate or describe activities that were not well known to those EPs who participated in OEF-A and OIF. Through mentioning specific branches of service, there is no intention to minimize the contributions of any other branch of service.

## CONCLUSION

OEF-A and OIF were the first major combat operations with robust EP participation. EPs’ unique skill sets served casualties well in combat’s highly varied environments: from the point-of-injury to flying ICUs. During the past 15 years, EPs led military medical units, participated in medical planning and engagement, became one of the military’s most deployed specialties, and provided invaluable battlefield trauma and medical care for one of the first times at this level in U.S. military history. Through research, civilian trainings based on the military experience (i.e., TCCC), and through daily clinical practice, the lessons learned in combat by EPs now shape the civilian practice environment.^33^
